# Problematic social media use: Conceptualization, assessment and trends in scientific literature

**DOI:** 10.1016/j.abrep.2020.100281

**Published:** 2020-05-05

**Authors:** Silvia Casale

**Affiliations:** Department of Health Sciences, University of Florence, Italy

**Keywords:** Problematic social media use, Social media addiction, Conceptualization, Classification, Assessment

## 1 Introduction

The rapid growth in popularity of social media has led to various theoretical discussions and empirical investigations on the potential benefits of their use. Social media represents an important developmental context for the handling of issues which are characteristic of adolescence such as gender and identity exploration, self-expression, and the increased need of peer acceptance (Gerwin, Kaliebe, & Daigle, 2018). Social media also allows users to overcome permanent real-life issues (e.g., physical disabilities) and age or work-related barriers (e.g., geographical distance to family members), helping some adults to satisfy the need to maintain social contact. Despite various advantages, evidence indicating some dysfunctional qualities of social media use have also stimulated hypotheses regarding the possibility that frequent use might hide a behavioral addiction (Müller et al., 2016). Other authors argue against this perspective by suggesting that an excessive use of social media might be an expression of pre-existing psychopathology (e.g., social anxiety or depression) rather than primary disorders (Shaffer, Hall, & Vander Bilt, 2000). Finally, some authors recommend a dose of skepticism towards the idea that frequent social media use might indicate a disorder or even only a mere symptom of a different primary condition, suggesting to systematically assess motivations for use as these might reflect a temporary compensatory strategy to cope with transient negative states (Kardefelt-Winther, 2014). These conflicting positions, however, converge upon the fact that an excessive use of social media, which we can term problematic social media use (PSMU), can have negative consequences in daily life. The purpose of this special issue is to provide a forum for the presentation of contemporary research on PSMU with the hope to help readers to identify promising areas for future research and clinical advances.

### 1.1 Overview of contents

The present special issue includes articles reporting original research findings as well as review articles on key topics in the field of PSMU. Three major themes are represented in this special issue: (1) the role of personality factors; (2) the role of social context; and (3) the examination of cognitive factors.

Sindermann and colleagues investigated personality differences between users and non-users of Facebook and the associations between personality and the overuse of Facebook (i.e. problematic Facebook use; PFU). Drawing on a very large community sample (N = 3835), they found that Facebook users reported higher levels of extraversion and lower levels of conscientiousness compared to non-users. They also found a significant association between PFU, on one hand, and conscientiousness and neuroticism, on the other, which was negative and positive, respectively. They call for more research on specific Facebook functions as it remains unclear if the personality factors identified were associated with problematic use towards all functions of Facebook or only toward specific functions.

Three studies focused on the potential contribution of narcissism, which has received growing scientific attention in this field, as this personality trait might be responsible for PSMU because of the need of narcissists to obtain admiration from wide audiences. Brailovskaya and colleagues added to previous findings by showing that the positive relationship between narcissism and PFU is mediated by the level of flow experienced on Facebook. Boursier and colleagues focused their attention on selfie behavior as a potential dysfunctional behavior when related to an objectified use of body images via social media. Their study found a positive association between two forms of narcissism (i.e. grandiosity and vulnerability) and selfie-engagement on social media and suggested that this association might be explained by body objectification. Casale and Banchi provided the first systematic review on the association between grandiose and vulnerable narcissism, on one hand, and PSMU, on the other hand. They found that narcissism might be involved in PFU, but it might not have consistent effects across social media platforms considering that studies that did not distinguish between different social media reported inconsistent results. They call for more research on specific platforms and suggest paying attention to potential moderators of the relationship.

The effect of parents and friends’ social media use is an understudied and potentially important area of research. By focusing their attention on the influence of friends (i.e., their social media use and group norms about social media use) Marino and colleagues found that friends’ social media use was associated with the frequency of one’s own social media use, which, in turn, was associated with problematic use. Their results also suggest that the crucial process associated with PSMU is the emphasis on expressing emotions on social media as a preferential tool for overcoming difficulties or strengthening relationships (i.e., “facilitating use of e-motions”), rather than a mere expression of emotions online. They suggest that this could cultivate a vicious cycle in which the unique benefit of social media use for emotion regulation is sustained and reinforces the belief of not being able to cope with emotions, thoughts, and decisions offline. The intergenerational transmission of PSMU was considered by Ruggeri and colleagues, from a different logic than what has been observed so far in the literature. By using an actor-partner interdependence model, the authors found support to their hypothesis that PFU in mothers affects the development of social anxiety concerning social media use in their offspring. Furthermore, they found a positive association between mothers’ PFU and adolescents’ PFU. Results from these two studies inform us that the influence of features of adolescents’ social context merits further investigation.

Since problematic smartphone users tend to spend more time on social networking or communication as opposed to other activities, Ryding and Kuss reviewed the passive objective measures that have been developed to assess problematic smartphone use. They highlighted the advantages of utilising objective passive monitoring within smartphone use research, on the one hand, and the numerous challenges associated with this type of assessment, on the other. Pertinent to the present special issue, their review supported that the Facebook app is the most frequently engaged with by problematic smartphone users.

Fioravanti and colleagues examined some key cognitive mechanisms driving PSMU, with a special emphasis on the dysfunctional beliefs included in the cognitive-behavioral model of Problematic Internet Use proposed by Davis (2001). One of their central findings is that maladaptive cognitions about the self (i.e. perfectionism discrepancies) and the world (i.e. social hopelessness) might predispose the user to the development of a preference for online social interactions.

### 1.2 Concluding remarks and future directions

While it is not the objective of this editorial to critically review the construct and clinical validity of PSMU, let me comment on some global concerns. The vast majority of the studies in the present field – including those presented in this special issue – tend to adopt a confirmatory approach in which PSMU is a priori considered as an addictive behavior. However, various core symptoms of addiction (e.g., mood modification, tolerance, and withdrawal symptoms; see Griffiths, 2005) have yet to be demonstrated. Substance addictions involve direct contact with brain synapses through the introduction of an exogenous ligand. In the behavioral addictions field, the neurotransmitter release may become dependent on repeated engagement in the behavior, which might lead to ‘withdrawal-like’ symptoms when the behavior is abruptly ceased (Sussman, 2017). In the PSMU field this perspective poses various issues. First, the empirical efforts that have been made to show neurobiological similarities between PSMU and established addictive behaviors have lead to conflicting results, especially regarding the activation of the prefrontal cortex (inhibition) brain system (He et al., 2017, He et al., 2017, Montag et al., 2017, Turel et al., 2014). Second, it has never been demonstrated that the object of the eventual addiction is *essential* to alleviate the negative states. This implies that the chance for the individual to use different strategies to regulate negative emotions might be maintained. Third, results about the experience of “withdrawal like symptoms” when the use of social media is interrupted are conflicting (Fernandez, Kuss, & Griffiths, 2020), with some studies showing withdrawal-like effects (Baumer et al., 2015, Sheldon et al., 2011, Stieger and Lewetz, 2018, Turel and Cavagnaro, 2018) and other studies highlighting that those who excessively use online social media seem to benefit from short breaks in terms of subjective well-being and perceived stress (Casale et al., 2019, Tromholt, 2016, Turel et al., 2018). Finally, one of the core factors helping to distinguish between normal and pathological conditions (the stability of the dysfunctional thoughts, emotions and behaviors over time) has yet to be empirically supported because of the paucity of longitudinal studies.

In conclusion, various major needs emerged. First, there is a need to demonstrate that PSMU is real: that is, it is a human experience that exists outside of any particular theoretical framework of PSMU. Psychological constructs are not objectively existing constituents of reality but rather “fictional” things that social scientists need to create in order to represent it (Billig, 2011). Second, if PSMU exists, there is a need to obtain more empirical evidence on its construct validity as a behavioral addiction. Moving away from a confirmatory approach would help to identify specific psychological precursors related to excessive social media use, which in turn would help to clarify the pathways implicated in the development of different behavioral addictions.
